# Analysis report on trends in public infectious disease control in China

**DOI:** 10.3389/fpubh.2024.1423191

**Published:** 2025-01-07

**Authors:** Zhaoting Zhang

**Affiliations:** School of Public Policy and Management, China University of Mining and Technology, Xuzhou, China

**Keywords:** infectious disease, global health, incidence rate, prevention, death rate, mortality

## Abstract

**Background:**

The prevention and control of public infectious diseases is a significant issue in the global health sector. Controlling infectious diseases is crucial for maintaining public health. As the most populous country in the world, China still faces a series of new challenges in the control of public infectious diseases. Therefore, it is of great significance to conduct an in-depth analysis of the trends in the control of public infectious diseases.

**Methodology:**

This study selects the death rate, incidence rate, proportion of prevention and control funds input, and the proportion of professional technical personnel in China from 2018 to 2023 as research samples and conducts statistical analysis through multiple linear regression. Overall, factors such as the incidence rate, proportion of prevention and control funds input, and proportion of professional technical personnel can explain 98.7% of the trend changes in the infectious disease death rate.

**Results:**

Through multiple regression analysis, the regression coefficient value of 0.001 for the incidence rate indicates a significant positive impact on the mortality rate, meaning that an increase in the incidence of infectious diseases leads to a rise in mortality. The regression coefficient value of −0.012 for the proportion of funding input suggests a significant negative impact on the mortality rate, implying that increased investment in prevention and control funds will correspondingly reduce the mortality rate of infectious diseases. On the other hand, merely increasing the number of professional and technical personnel is not sufficient to control the spread of infectious diseases; comprehensive use of various prevention and control measures is required for effective public infectious disease control.

**Conclusion:**

Public infectious disease prevention and control is a complex process that requires the consideration of multiple factors, rather than merely changing a single factor, particularly in controlling incidence rates and reasonably allocating funds. By refining the analysis of infectious disease control strategies and integrating diverse preventive and intervention measures, it is possible to better control the spread and mortality of infectious diseases, thereby protecting public health and safety.

## 1 Introduction

**Figure 1:**
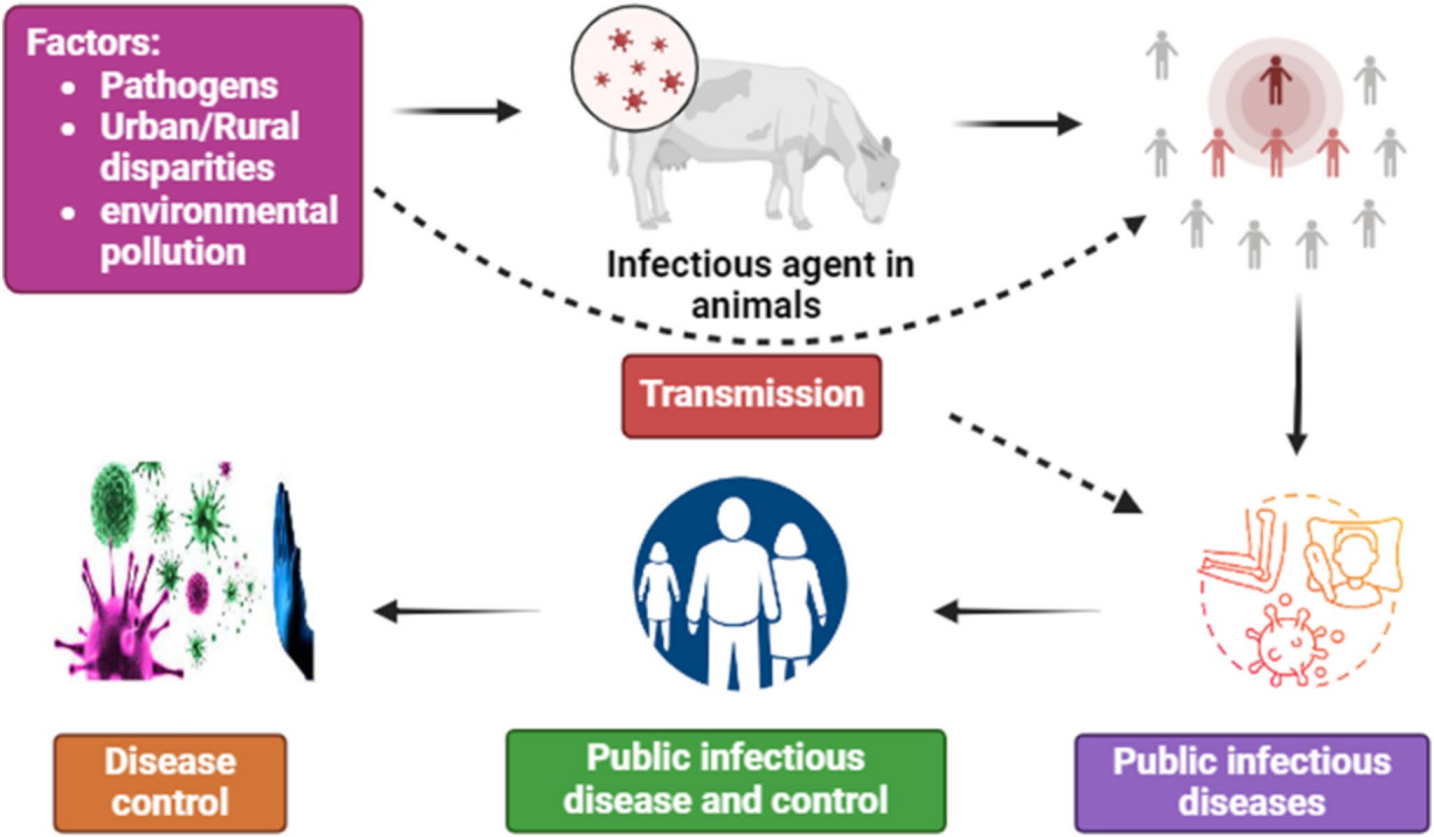
Prevalence of public infectious disease and control.

Public infectious disease prevention and control is a crucial issue in the global health sector, as controlling infectious diseases is paramount for maintaining public health (1). China, as one of the most populous countries in the world, directly relates its public health security and social stability to the control of public infectious diseases. With the acceleration of globalization and the increase in population mobility, the world faces various threats and challenges from infectious diseases such as influenza, tuberculosis, HIV/AIDS, avian influenza, etc. (2). In the past few decades, China has made significant progress in the field of public health by establishing sound monitoring systems, increasing investment in prevention, vaccination, improving health facilities, and enhancing international cooperation, successfully controlling the spread and outbreaks of various infectious diseases (3). However, with factors such as the spread of pathogens, urban–rural disparities, and environmental pollution, China still faces a series of new challenges, such as inadequate vaccination rates and increasing antibiotic resistance (4, 5). Therefore, conducting in-depth analysis of the trend of public infectious disease control is of great significance. Public infectious disease control trends refer to the development direction or trends presented in the control and management of infectious diseases in a specific region or country. The purpose of public infectious disease trend control is to reduce the incidence and mortality rates of infectious diseases. These trends involve various aspects, including the mortality rate, incidence rate, investment in prevention and control costs, transmission routes, and utilization of medical resources. Analysis of public infectious disease control trends can help governments, health institutions, and researchers better understand the dynamic changes of infectious diseases. This not only helps in comprehensively understanding the current public health challenges and issues but also aids in proposing control policy recommendations and prevention strategies concerning the development trends of infectious diseases (6). According to the medical community’s definition of the nature of infectious diseases, infectious diseases are a class of diseases caused by various pathogens that can be transmitted between humans, animals, or between humans and animals (Figure 1) (7). According to the provisions of the Infectious Diseases Prevention and Control Law of the People’s Republic of China, infectious diseases are classified into three categories (A, B, and C) based on the outbreak, prevalence, and degree of harm when they occur (8). Category A infectious diseases include plague and cholera; Category B infectious diseases include infectious atypical pneumonia, AIDS, viral hepatitis, poliomyelitis, human infection with highly pathogenic avian influenza, measles, epidemic hemorrhagic fever, rabies, epidemic encephalitis B, dengue fever, anthrax, bacterial and amoebic dysentery, tuberculosis, typhoid and paratyphoid fever, epidemic meningitis, pertussis, diphtheria, neonatal tetanus, scarlet fever, brucellosis, gonorrhea, syphilis, leptospirosis, schistosomiasis, and malaria; Category C infectious diseases include influenza, epidemic parotitis, rubella, acute hemorrhagic conjunctivitis, leprosy, epidemic and endemic typhoid and paratyphoid fever, black fever, hydatid disease, and filariasis, except for cholera, bacterial and amoebic dysentery, and typhoid and paratyphoid fever. Other infectious diseases outside the above catalog, according to their outbreak, prevalence, and degree of harm, need to be classified as category B or C infectious diseases, which are determined and announced by the health administrative department of the State Council of China (8).

This article collected and analyzed the number of cases and deaths of category A, B, and C infectious diseases in China since 2018, as well as the government’s financial expenditure on the control of major public infectious diseases and the number of professional and technical personnel for prevention and control of infectious diseases. It analyzed the trend of public infectious diseases and their control and conducted multivariate linear regression analysis on relevant factors. The purpose of this article is to emphasize the critical role of public infectious disease prevention and control in maintaining public health and social stability through a study of the correlation trends in public infectious disease control. It aims to provide governments, health institutions, and researchers with deeper insights to formulate more targeted control policies and methods, thereby helping to enhance China’s public health response capabilities to better address the threats posed by infectious diseases and ensure the health and stability of the public. This is also the contribution of this article.

## 2 Literature review

The analysis of trends in public infectious disease control is an important research field involving multiple disciplines such as public health, epidemiology, and medicine (9). The theoretical foundation of research on trends in public infectious disease control involves various disciplines and theoretical frameworks, mainly including epidemiological theory, behavioral science theory, and health services management theory (10). Epidemiological theory views public infectious diseases and other epidemiological phenomena as the study of disease spread and impact within populations. Through epidemiological investigations and analysis, it can reveal the patterns, transmission routes, and high-risk populations of public infectious disease outbreaks, providing a scientific basis for the development of effective strategies to control disease transmission (11). Behavioral science theory focuses on individual and collective behavior patterns, preferences, and decisions. This theoretical perspective is crucial for understanding people’s behavioral responses and health decisions in the context of infectious disease transmission control. Behavioral science theory can be used to analyze the public’s cognition, attitudes, and behaviors regarding infectious disease trends, guiding the design of effective health education and control strategies (12). Health services management theory involves aspects such as the organization, management, policy formulation, and resource allocation of health systems. It is essential for planning, implementing, and evaluating the control of public infectious disease trends. Health services management theory can be utilized to assess the responsiveness of infectious disease control systems, the effectiveness of trend control policies, and the fairness of medical resource allocation (13). It can be observed that the theoretical foundation of research on trends in public infectious disease control encompasses multiple theoretical perspectives. It requires the comprehensive application of epidemiology, behavioral science, health services management, sociology, and information technology to comprehensively and systematically understand and address the challenges faced in controlling trends in public infectious diseases.

Both internationally and domestically in China, many scholars have conducted research and analysis on the trends in public infectious disease control. The research encompasses various aspects, typically covering the processes, challenges, strategies, and trends in infectious disease trend control over the past, present, and future. These studies include: “Infectious disease in an era of global change” by Rachel E. Bake et al. This paper analyzes the epidemiological trends of major infectious diseases globally, including HIV/AIDS, tuberculosis, malaria, and influenza. It summarizes the transmission patterns, influencing factors, and effectiveness of control strategies for these diseases, providing reference for the formulation of global infectious disease control policies (14). “Mathematical modeling of infectious disease dynamics” by Constantinos I. Siettos and Lucia Russo. This paper introduces the application of mathematical modeling and simulation in infectious disease control. By constructing mathematical models of disease transmission and utilizing computer simulation techniques, researchers can evaluate the effects of different intervention measures, optimize prevention and control strategies, and predict the development trends of epidemics, thus providing scientific basis for decision-making in disease control (15). “Global trends in emerging infectious diseases” by Kate E. Jones et al. This paper focuses on analyzing the global trends and challenges of emerging infectious diseases, including the impact of pathogen variation, population mobility, and globalization on disease transmission. It emphasizes the need for closer global cooperation to address the challenges posed by emerging infectious diseases (16). “Emphasis of infection prevention and control: A review” by Harsh Thakur and Rahul Rao. This paper reviews the trends and prospects of infectious disease prevention and control measures, including innovations in funding, vaccine development, drug treatment, and rapid testing technology. It suggests that with continuous technological advancements, infectious disease prevention and control will become more intelligent and precise, offering new possibilities for achieving comprehensive disease control (17). In China, the article “China’s 70-year Effectiveness in the Prevention and Control of Infectious Diseases” by Yang Weizhong analyzes the prevention and control processes of infectious diseases such as smallpox, plague, cholera, dysentery, typhoid fever, measles, diphtheria, pertussis, meningococcal meningitis, mumps, schistosomiasis, and hemorrhagic fever over the past 70 years. It significantly reduced the incidence and mortality of infectious diseases, providing confidence and insights for future disease prevention and control efforts (18). Additionally, Zhou Yuhui’s article “Research on the Current Situation of Infectious Diseases and the Construction of Prevention and Control System in China” comprehensively reviews the epidemic situation of infectious diseases in China and systematically analyzes the prevention and control system from aspects such as legal and regulatory framework, joint prevention and control mechanism, prevention and control institutions, monitoring and early warning system, vaccine research and development, and fundraising mechanism. The article proposes recommendations for the development of a modernized infectious disease prevention and control system from the perspectives of comprehensive prevention and control strategies, monitoring and early warning capabilities, increased funding, enhanced technological innovation support, and strengthened international communication and cooperation (19). Although these literature pieces analyze the development and measures of infectious disease prevention and control from various perspectives, they lack more detailed data analysis on the correlation between the incidence, mortality, and control measures of infectious diseases in specific regions and countries. This paper aims to study the changing trends in the incidence and mortality of infectious diseases in China in recent years and analyze the linear relationship between infectious disease mortality rate and control measures using multivariate linear regression methods. The goal is to explore the trends in infectious disease control and provide insights and measures for future epidemic trend control, which is the purpose of this study.

## 3 Data and methodology

### 3.1 Data

The People’s Republic of China established the Ministry of Health in 1952 and set up institutions for the prevention and control of infectious diseases and epidemiological research, strengthening the monitoring and control of infectious diseases. In 1955, China successfully controlled a large-scale outbreak of plague (20). Meanwhile, China gradually established a surveillance and reporting system for infectious diseases, forming a four-level disease monitoring network at the national, provincial, municipal, and county levels. Disease monitoring and reporting have become important means of prevention and control, helping to detect outbreaks promptly (21). From the late 20th century to the early 21st century, China faced a series of severe infectious disease outbreaks, such as Severe Acute Respiratory Syndrome (SARS) and avian influenza. These outbreaks posed new challenges to China’s infectious disease prevention and control efforts and prompted the government to take more proactive measures to strengthen the prevention and control system for infectious diseases and enhance its capacity to respond to public health emergencies (22). This study selected the trend changes in the mortality rate and incidence proportion of infectious diseases in China from 2018 to 2023 as the research sample. Due to the wide scope of the outbreak of novel coronavirus infections, government special funding and separate plans were implemented for prevention and control, so they were not included in the analysis scope of this study.

In 2018, there were 3.063 million reported cases and 23,174 reported deaths from Class A and B infectious diseases nationwide in China. The top five reported diseases by incidence were viral hepatitis, pulmonary tuberculosis, syphilis, gonorrhea, and bacterial and amoebic dysentery, accounting for 92.2% of the total reported cases of Class A and B infectious diseases. The top five reported diseases by mortality were AIDS, pulmonary tuberculosis, viral hepatitis, rabies, and Japanese encephalitis, representing 99.3% of the total reported deaths from Class A and B infectious diseases. In 2018, the mainland population of China was 1,395.38 million (excluding Hong Kong, Macao, and Taiwan). Calculated per 100,000 people, the incidence rate of Class A and B infectious diseases nationwide was 219.51/100,000, and the mortality rate was 1.66/100,000 (23). In 2019, there were 3.072 million reported cases and 24,981 reported deaths from Class A and B infectious diseases nationwide in China. The top five reported diseases by incidence were viral hepatitis, pulmonary tuberculosis, syphilis, gonorrhea, and scarlet fever, accounting for 91.1% of the total reported cases of Class A and B infectious diseases. The top five reported diseases by mortality were AIDS, pulmonary tuberculosis, viral hepatitis, rabies, and epidemic hemorrhagic fever, representing 99.6% of the total reported deaths from Class A and B infectious diseases. In 2019, the mainland population of China was 1,400.05 million (excluding Hong Kong, Macao, and Taiwan). Calculated per 100,000 people, the incidence rate of Class A and B infectious diseases nationwide was 219.44/100,000, and the mortality rate was 1.78/100,000 (24). In 2020, there were 2.58 million reported cases and 21,655 reported deaths from Class A and B infectious diseases nationwide in China. The top five reported diseases by incidence were viral hepatitis, pulmonary tuberculosis, syphilis, gonorrhea, and AIDS, accounting for 94.39% of the total reported cases of Class A and B infectious diseases. The top five reported diseases by mortality were AIDS, pulmonary tuberculosis, viral hepatitis, rabies, and syphilis, representing 99.5% of the total reported deaths from Class A and B infectious diseases. In 2020, the mainland population of China was 1,411.77 million (excluding Hong Kong, Macao, and Taiwan). Calculated per 100,000 people, the incidence rate of Class A and B infectious diseases nationwide was 183.19/100,000, and the mortality rate was 1.53/100,000 (25). In 2021, there were 2.712 million reported cases and 22,177 reported deaths from Class A and B infectious diseases nationwide in China. The top five reported diseases by incidence were viral hepatitis, pulmonary tuberculosis, syphilis, gonorrhea, and brucellosis, accounting for 93.3% of the total reported cases of Class A and B infectious diseases. The top five reported diseases by mortality were AIDS, pulmonary tuberculosis, viral hepatitis, rabies, and epidemic hemorrhagic fever, representing 99.7% of the total reported deaths from Class A and B infectious diseases. In 2021, the mainland population of China was 1,412.60 million (excluding Hong Kong, Macao, and Taiwan). Calculated per 100,000 people, the incidence rate of Class A and B infectious diseases nationwide was 191.98/100,000, and the mortality rate was 1.57/100,000 (26). In 2022, there were 2.431 million reported cases and 21,834 reported deaths from Class A and B infectious diseases nationwide in China (excluding novel coronavirus infections). The top five reported diseases by incidence were viral hepatitis, pulmonary tuberculosis, syphilis, gonorrhea, and brucellosis, accounting for 93.4% of the total reported cases of Class A and B infectious diseases. The top five reported diseases by mortality were AIDS, pulmonary tuberculosis, viral hepatitis, rabies, and epidemic hemorrhagic fever, representing 99.8% of the total reported deaths from Class A and B infectious diseases. In 2022, the mainland population of China was 1,411.75 million (excluding Hong Kong, Macao, and Taiwan). Calculated per 100,000 people, the incidence rate of Class A and B infectious diseases nationwide was 172.22/100,000, and the mortality rate was 1.55/100,000 (27). In 2023, there were 3.5078 million reported cases and 25,525 reported deaths from Class A and B infectious diseases nationwide in China (excluding novel coronavirus infections). The top five reported diseases by incidence were viral hepatitis, pulmonary tuberculosis, syphilis, gonorrhea, and brucellosis, accounting for 92.5% of the total reported cases of Class A and B infectious diseases. The top five reported diseases by mortality were AIDS, pulmonary tuberculosis, viral hepatitis, rabies, and syphilis, representing 99.8% of the total reported deaths from Class A and B infectious diseases. In 2023, the mainland population of China was 1,409.67 million (excluding Hong Kong, Macao, and Taiwan). Calculated per 100,000 people, the incidence rate of Class A and B infectious diseases nationwide was 248.83/100,000, and the mortality rate was 1.81/100,000 (28).

specific data can be found in Table 1.

**Table 1:** Statistics of the incidence and mortality of class A and B public infectious diseases in China from 2018 to 2023.

Project/year	Number of cases in million	Number of deaths	Population in million	Incidence rate/100,000	Death rate/100,000
2018	3.063	23,174	1395.38	219.51	1.66
2019	3.072	24,981	1400.05	219.44	1.78
2020	2.58	21,655	1411.77	183.19	1.53
2021	2.712	22,177	1412.60	191.98	1.57
2022	2.431	21,834	1411.75	172.22	1.55
2023	3.5078	25,525	1409.67	248.83	1.81

Data source: Chinese Health Commission and Chinese National Administration of Disease Control and Prevention website (23–28).

Overall, from 2018 to 2023, the reported incidence and mortality of Class A and B infectious diseases nationwide showed a downward trend (Table 1). However, in 2023, due to the emergence of monkeypox as a Class B contagious disease, there was an upward trend in the reported incidence of Class B infectious diseases.

In 2018, there were a total of 4.708 million reported cases and 203 deaths from Class C infectious diseases nationwide in China. The top five reported diseases by incidence were hand, foot, and mouth disease; other infectious diarrheal diseases; influenza; epidemic parotitis; and acute hemorrhagic conjunctivitis, accounting for 99.8% of the total reported cases of Class C infectious diseases. The diseases with the highest number of reported deaths were influenza, hand, foot, and mouth disease, and other infectious diarrheal diseases, representing 100% of the total reported deaths from Class C infectious diseases. In 2018, the mainland population of China was 1,395.38 million (excluding Hong Kong, Macao, and Taiwan). Calculated per 100,000 people, the incidence rate of Class C infectious diseases nationwide was 337.38/100,000, and the mortality rate was 0.0146/100,000 (23). In 2019, there were a total of 7.172 million reported cases and 304 deaths from Class C infectious diseases nationwide in China. The top five reported diseases by incidence were influenza, hand, foot, and mouth disease; other infectious diarrheal diseases; epidemic parotitis; and acute hemorrhagic conjunctivitis, accounting for 99.5% of the total reported cases of Class C infectious diseases. The diseases with the highest number of reported deaths were influenza, hand, foot, and mouth disease, and other infectious diarrheal diseases, representing 99.3% of the total reported deaths from Class C infectious diseases. In 2019, the mainland population of China was 1,400.05 million (excluding Hong Kong, Macao, and Taiwan). Calculated per 100,000 people, the incidence rate of Class C infectious diseases nationwide was 512.28/100,000, and the mortality rate was 0.021/100,000 (24). In 2020, there were a total of 3.13 million reported cases and 85 deaths from Class C infectious diseases nationwide in China. The top five reported diseases by incidence were influenza; other infectious diarrheal diseases; hand, foot, and mouth disease; epidemic parotitis; and acute hemorrhagic conjunctivitis, accounting for 94.7% of the total reported cases of Class C infectious diseases. The diseases with the highest number of reported deaths were influenza, other infectious diarrheal diseases, and hand, foot, and mouth disease, representing 96.5% of the total reported deaths from Class C infectious diseases. In 2020, the mainland population of China was 1,411.77 million (excluding Hong Kong, Macao, and Taiwan). Calculated per 100,000 people, the incidence rate of Class C infectious diseases nationwide was 221.95/100,000, and the mortality rate was 0.0061/100,000 (25). In 2021, there were a total of 3.506 million reported cases and 19 deaths from Class C infectious diseases nationwide in China. The top five reported diseases by incidence were hand, foot, and mouth disease; other infectious diarrheal diseases; influenza; epidemic parotitis; and acute hemorrhagic conjunctivitis, accounting for 99.9% of the total reported cases of Class C infectious diseases. The diseases with the highest number of reported deaths were hand, foot, and mouth disease; other infectious diarrheal diseases; and influenza, representing 94.7% of the total reported deaths from Class C infectious diseases. In 2021, the mainland population of China was 1,412.60 million (excluding Hong Kong, Macao, and Taiwan). Calculated per 100,000 people, the incidence rate of Class C infectious diseases nationwide was 248.22/100,000, and the mortality rate was 0.0013/100,000 (26). In 2022, there were a total of 4.210 million reported cases and 27 deaths from Class C infectious diseases nationwide in China. The top five reported diseases by incidence were influenza; other infectious diarrheal diseases; hand, foot, and mouth disease; epidemic parotitis; and acute hemorrhagic conjunctivitis, accounting for 99.9% of the total reported cases of Class C infectious diseases. The diseases with the highest number of reported deaths were influenza, other infectious diarrheal diseases, and hand, foot, and mouth disease, representing 92.6% of the total reported deaths from Class C infectious diseases. In 2022, the mainland population of China was 1,411.75 million (excluding Hong Kong, Macao, and Taiwan). Calculated per 100,000 people, the incidence rate of Class C infectious diseases nationwide was 298.23/100,000, and the mortality rate was 0.0019/100,000 (27). In 2023, there were a total of 15.6618 million reported cases and 86 deaths from Class C infectious diseases nationwide in China. The top five reported diseases by incidence were influenza; other infectious diarrheal diseases; hand, foot, and mouth disease; epidemic parotitis; and acute hemorrhagic conjunctivitis, accounting for 96.6% of the total reported cases of Class C infectious diseases. The diseases with the highest number of reported deaths were influenza and dengue fever, representing 98.8% of the total reported deaths from Class C infectious diseases. In 2023, the mainland population of China was 1,409.67 million (excluding Hong Kong, Macao, and Taiwan). Calculated per 100,000 people, the incidence rate of Class C infectious diseases nationwide was 1,111.03/100,000, and the mortality rate was 0.006/100,000 (28). Overall, from 2019 to 2022, the reported incidence and mortality of Class C infectious diseases nationwide showed a downward trend. However, in 2023, due to mutations in the coronavirus and other factors such as climate, there was a significant increase in influenza (29).

Specific data can be found in Tables 2, 3.

**Table 2:** Statistics of the incidence and mortality of class C public infectious diseases in China from 2018 to 2023.

Project\year	Number of cases in million	Number of deaths	Population in million	Incidence rate/100,000	Death rate/100,000
2018	4.708	203	1395.38	337.38	0.0146
2019	7.172	304	1400.05	512.28	0.021
2020	3.13	85	1411.77	221.95	0.0061
2021	3.506	19	1412.6	248.22	0.0013
2022	4.21	27	1411.75	298.23	0.0019
2023	15.6618	86	1409.67	1111.03	0.006

Data source: Chinese Health Commission and Chinese National Administration of Disease Control and Prevention website (23–28).

**Table 3:** Number of reported incidences and deaths of class C infectious diseases nationwide from 2018 to 2023.

Disease name	Number of cases						Death toll					
	2018	2019	2020	2021	2022	2023	2018	2019	2020	2021	2022	2023
Influenza	765,186	3,538,213	1,145,278	668,246	2,442,797	12,515,250	153	269	70	4	13	81
Mumps	259,071	299,961	129,120	119,955	104,016	89,642			1			
Rubella	3,930	32,539	2,201	840	784	2,118					1	
Acute hemorrhagic conjunctivitis	38,250	41,439	28,471	28,350	26,097	197,041						
Leprosy	225	233	200	180	143	372						
Typhus	971	1,173	1,069	1,310	1,291	1,665						
Kala azar	160	151	202	230	226	544			1		1	4
Hydatid disease	4,327	4,003	3,327	2,799	2,341	3,724		2	1	1		
Filariasis				1		42,950						
Other infectious diarrhea diseases	1,282,270	1,335,627	1,062,277	1,329,790	959,636	1,128,115	15	13	9	6	6	
Hand foot and mouth disease	2,353,310	1,918,830	761,355	1,354,548	672,911	1,680,376	35	20	3	8	6	1
Total	4,707,700	7,172,169	3,133,500	3,506,249	4,210,242	15,661,797	203	304	85	19	27	86

Unit: individual. Data source: Chinese Health Commission and Chinese National Administration of Disease Control and Prevention website (23–28).

Here’s the summary of the reported incidence and mortality rates of Class A, B, and C infectious diseases per 100,000 people: In 2018, the nationwide reported incidence rate of Class A, B, and C infectious diseases combined was 556.89/100,000, and the combined mortality rate was 1.67/100,000; In 2019, the nationwide reported incidence rate of Class A, B, and C infectious diseases combined was 731.72/100,000, and the combined mortality rate was 1.81/100,000; In 2020, the nationwide reported incidence rate of Class A, B, and C infectious diseases combined was 405.14/100,000, and the combined mortality rate was 1.54/100,000; in 2021, the nationwide reported incidence rate of Class A, B, and C infectious diseases combined was 440.2/100,000, and the combined mortality rate was 1.57/100,000; in 2022, the nationwide reported incidence rate of Class A, B, and C infectious diseases combined was 470.45/100,000, and the combined mortality rate was 1.55/100,000. In 2023, the nationwide reported incidence rate of Class A, B, and C infectious diseases combined was 1359.86/100,000, and the combined mortality rate was 1.82/100,000. Specific data can be found in Table 4.

**Table 4:** Statistics of total cases and deaths of class A, B and C public infectious diseases from 2018 to 2023.

Years\Project	2018	2019	2020	2021	2022	2023
Incidence rate/100,000	556.89	731.72	405.14	440.2	470.45	1359.86
Death rate/100,000	1.67	1.81	1.54	1.57	1.55	1.82

Data source: Chinese Health Commission and Chinese National Administration of Disease Control and Prevention website (23–28).

Public infectious disease prevention and control involves various measures to prevent the spread and outbreak of infectious diseases in populations, thereby reducing mortality rates and ensuring public health security. Common measures for public infectious disease prevention and control include government investment in prevention and control funding, monitoring and early warning by professional technical personnel, vaccination, health education and public awareness campaigns, medical interventions, and more. Although vaccines and vaccination are among the most cost-effective public health interventions (30). However, due to the variety of vaccine types and the multitude of vaccination providers, the total volume and coverage rate of vaccine administration for infectious diseases cannot be accurately measured. Promotion, public awareness, and medical interventions are subject to the scope and technical proficiency, making quantitative comparisons challenging. Government investment in prevention and control funding and the number of technical personnel in specialized infectious disease prevention and control institutions can be quantified. Therefore, this study selects government investment in prevention and control funding and the number of technical personnel in specialized prevention and control institutions as the analytical sample for infectious disease control measures. On the one hand, these two measures are directly related to the trends in infectious disease prevention and control. On the other hand, the data for these two measures can be analyzed quantitatively.

From 2018 to 2023, the Chinese government consistently provided special subsidies for major infectious disease prevention and control funding. The subsidy funds were mainly used for expanding efforts in the prevention and treatment of infectious diseases and other relevant major public health services, and were incorporated into the government’s annual budget expenditures. In 2018, the investment in prevention and control funding amounted to 16981.77 billion RMB, for every 100,000 people, the funding allocation is 1,217,000 RMB (31). In 2019, the investment in prevention and control funding reached 16525.61 billion RMB, for every 100,000 people, the funding allocation is 1,180,400 RMB (32). In 2020, the investment in prevention and control funding was 17525.61 billion RMB, for every 100,000 people, the funding allocation is 1,241,400 RMB (33). In 2021, the investment in prevention and control funding totaled 19212.08 billion RMB, for every 100,000 people, the funding allocation is 1,360,100 RMB (34). In 2022, the investment in prevention and control funding rose to 20379.58 billion RMB, for every 100,000 people, the funding allocation is 1,443,600 RMB (35). In 2023, the investment in prevention and control funding increased to 23820.01 billion RMB, for every 100,000 people, the funding allocation is 1,689,800 RMB (36). It can be observed that the amount and proportion of investment in prevention and control funding have shown an increasing trend annually from 2018 to 2023. Specific data can be found in Table 5.

**Table 5:** Investment of prevention and control funds from 2018 to 2023.

Year\project	2018	2019	2020	2021	2022	2023	Remark
Prevention and control funds	16981.77	16525.61	17525.61	19212.1	20379.58	23820.01	RMB million
Proportion of prevention and control funds	121.7	118.04	124.14	136.01	144.36	168.98	10000RMB/100,000 people

Data sources: China Ministry of Finance and China National Center for Disease Control and Prevention websites (31–36).

The Chinese government has specifically established around 3,400 professional disease prevention and control centers nationwide, separate from hospitals. The mission of these centers is to create a healthy environment and safeguard public health through the prevention and control of infectious diseases and other illnesses. Their responsibilities include formulating regulations, policies, standards, and prevention and control plans related to infectious diseases and public health; developing national implementation plans for the prevention and control of major infectious diseases and other illnesses; monitoring the development and distribution patterns of infectious diseases and proposing prevention and control strategies; participating in and guiding local responses to major epidemics and public health emergencies; and establishing emergency response systems for major public health issues such as major infectious diseases and disaster prevention and disease control.

The number of professional technical personnel in disease prevention and control institutions is closely related to the prevention and control of infectious diseases. In 2018, there were 140,000 professional technical personnel, accounting for 10.03 per 100,000 people (23). In 2019, there were 140,000 professional technical personnel, accounting for 9.99 per 100,000 people (24). In 2020, there were 145,000 professional technical personnel, accounting for 10.27 per 100,000 people (25). In 2021, there were 158,000 professional technical personnel, accounting for 11.19 per 100,000 people (26). In 2022, there were 169,000 professional technical personnel, accounting for 11.97 per 100,000 people (27). In 2023, there were 166,000 professional technical personnel, accounting for 11.76 per 100,000 people (28). As disease prevention and control institutions are not treatment facilities but rather research and management entities, the number of professional technical personnel does not need to be particularly large. It can be observed that the total number and proportion of professional technical personnel have shown an increasing trend annually from 2018 to 2023. For specific data, please refer to Table 6.

**Table 6:** Statistics of public infectious disease prevention and control technicians from 2018 to 2023.

Year\project	2018	2019	2020	2021	2022	2023	Remark
Number of technicians	14	14	14.5	15.8	16.9	16.6	Ten thousand
Proportion of technical personnel	10.03	9.99	10.27	11.19	11.97	11.76	/100,000 people

Data source: The data description of prevention and control technicians mentioned above (23–28).

### 3.2 Methodology

The content of this study focuses on the trend analysis of public infectious disease prevention and control. The research objective aims to control the spread and prevalence of infectious diseases within populations through relevant measures. There are two key indicators for infectious disease control: mortality rate control and incidence rate control (37). Among them, controlling the mortality rate of infectious disease infections is a crucial indicator.

Below, we employ multiple linear regression to analyze several factors related to infectious diseases: mortality rate, incidence rate, proportion of expenditure, and proportion of professional technical personnel. Specifically, we take the mortality rate of infectious diseases as the dependent variable (Y) and the incidence rate, proportion of expenditure, and proportion of professional technical personnel as independent variables (X) for multiple regression analysis. We select data from 2018 to 2023 for infectious disease mortality rate, incidence rate, proportion of expenditure, and proportion of professional technical personnel as the sample, with *N* = 6. All these samples are valid, with no invalid samples present. See Table 4 for specific data (see Table 7)

**Table 7:** Statistical table of the mortality rate, incidence rate, funding investment ratio, and professional and technical personnel ratio of infectious diseases per 100,000 people from 2019 to 2023.

Year\project	2018	2019	2020	2021	2022	2023
Mortality rate	1.67	1.81	1.54	1.57	1.55	1.82
Incidence	556.89	731.72	405.14	440.2	470.45	1359.86
Proportion of funding investment	121.7	118.04	124.14	136.01	144.36	168.98
Proportion of number of professional and technical personnel	10.03	9.99	10.27	11.19	11.97	11.76

Data source: summary of mortality, morbidity and other data indicators above.

This study conducts computations and analyses using SPSS (Statistical Package for the Social Sciences) software. The data from 2018 to 2023 for infectious disease mortality rate, incidence rate, proportion of expenditure, and proportion of professional technical personnel, as presented in the tables above, are analyzed using SPSS. Regression analysis begins with assessing the model fit, primarily through analyzing the R-square value to evaluate how well the model fits the data. Additionally, analysis of the Variance Inflation Factor (VIF) is conducted to determine if there are multicollinearity issues in the model. Subsequently, the regression model equation is derived. Next, the significance of the independent variables (X) is analyzed. If they exhibit significance (*p*-value less than 0.05 or 0.01), it indicates an influential relationship between X and the dependent variable (Y). The direction of this influence is also analyzed. Finally, a summary of the analysis is provided. The results of the regression analysis conducted through the SPSS system are presented in Table 8.

**Table 8:** Linear regression analysis results (*N* = 6).

Project	Unstandardized coefficient		Standardized coefficient	*T*	*P*	Collinearity diagnosis	
	B	Standard error	beta			VIF	Tolerance
Constant	1.576	0.182	-	8.654	0.013*	-	-
Incidence	0.001	0	1.831	8.533	0.013*	7.185	0.139
Funding investment ratio	−0.012	0.003	−1.767	−4.347	0.049*	25.772	0.039
Proportion of number of professional and technical personnel	0.117	0.044	0.805	2.68	0.116	14.077	0.071
*R* ^2^	0.987						
Adjustment*r*^2^	0.968						
F	F(3,2)=51.363, *p* = 0.019						
D-W value	3.026						

Parameter settings, dependent variable Y is: mortality rate, *p* < 0.05 *p* < 0.01.

After analysis, it is evident from the table that conducting a linear regression analysis with the incidence rate, proportion of expenditure, and proportion of professional technical personnel as independent variables (X), and mortality rate as the dependent variable (Y), yields the following model equation: Mortality Rate = 1.576 (constant) + 0.001 (regression coefficient B) * Incidence Rate (X1)–0.012 (regression coefficient B) * Proportion of Expenditure (X2) + 0.117 (regression coefficient B) * Proportion of Professional Technical Personnel (X3). The R-square value of the model is 0.987, indicating that 98.7% of the variance in mortality rate can be explained by the incidence rate, proportion of expenditure, and proportion of professional technical personnel. The model passed the F-test (*F* = 51.363, *p* = 0.019 < 0.05), indicating that at least one of the variables (incidence rate, proportion of expenditure, and proportion of professional technical personnel) has a significant impact on mortality rate. Additionally, multicollinearity was detected, as indicated by VIF values exceeding 10. Further analysis of the specific data reveals that the regression coefficient for the incidence rate is 0.001 (T = 8.533, *p* = 0.013 < 0.05), indicating a significant positive relationship between the incidence rate and mortality rate. The regression coefficient for the proportion of expenditure is −0.012 (T = −4.347, *p* = 0.049 < 0.05), indicating a significant negative relationship between the proportion of expenditure and mortality rate. However, the regression coefficient for the proportion of professional technical personnel is 0.117 (T = 2.680, *p* = 0.116 > 0.05), indicating that it does not significantly affect mortality rate. In summary, the analysis indicates that the incidence rate has a significant positive effect on mortality rate, while the proportion of expenditure has a significant negative effect on mortality rate. However, the proportion of professional technical personnel does not affect mortality rate.

Regarding the issue of using the F-test to determine the significance of a regression model, if the model passes the F-test (*p* < 0.05), it indicates that the model is meaningful, and at least one X variable influences Y. If the model fails the F-test (*p* > 0.05), it suggests that the model construction is not meaningful, and none of the X variables affect Y. According to the SPSS analysis results, specific data are shown in Table 9.

**Table 9:** ANOVA table (intermediate process).

Project	Sum of square	DF	Mean square	F	*p* value
Return	0.082	3	0.027	51.363	0.019
Residual	0.001	2	0.001		
Total	0.083	5			

The analysis results indicate that the model passes the *F*-test (*F* = 51.363, *P* = 0.019 < 0.05), which means that the model construction is meaningful.

## 4 Results

With the continuous improvement of China’s healthcare system and the strengthening of public health measures, the incidence rates of many infectious diseases have shown a declining trend. For example, the incidence rates of infectious hepatitis, tuberculosis, and AIDS have all decreased in recent years (38). However, overall, due to the impact of diseases such as seasonal flu, the mortality and incidence rates of infectious diseases have shown a fluctuating trend, with an increase in mortality rates and a significant increase in incidence rates in 2023.

To study the trends in public infectious disease prevention and control, this study selected several parameters including the mortality rate, incidence rate, proportion of expenditure, and proportion of professional technical personnel, analyzing methods to control the spread of infectious diseases. Through multiple regression analysis, the regression coefficient value of the incidence rate is 0.001 (T = 8.533, *p* = 0.013 < 0.05), indicating a significant positive impact of the incidence rate on the mortality rate. This suggests that an increase in the incidence rate of infectious diseases corresponds to an upward trend in mortality rates. The regression coefficient value of the expenditure ratio is −0.012 (T = −4.347, *p* = 0.049 < 0.05), meaning that the expenditure ratio has a significant negative impact on the mortality rate. An increase in expenditure on prevention and control leads to a decrease in the mortality rate of infectious diseases. The regression coefficient value of the proportion of professional technical personnel is 0.117 (T = 2.680, *p* = 0.116 > 0.05), indicating that the proportion of professional technical personnel does not have a significant impact on the mortality rate. This means that an increase in the number of professional technical personnel in infectious disease prevention and control institutions does not significantly control the decline in mortality rates, requiring the comprehensive use of other prevention and control measures. This study demonstrates that although there are many measures for infectious disease prevention and control, whether they can directly control the mortality rate of infectious diseases requires detailed analysis. Infectious disease prevention and control is the result of the application of comprehensive measures. The analysis of the model’s R-squared value of 0.987 indicates that the incidence rate, expenditure ratio, and proportion of professional technical personnel can explain 98.7% of the trend changes in mortality rates.

## 5 Discussion

In the 1950s, China had high incidence, prevalence, and mortality rates for infectious diseases. Severe infectious diseases such as plague, cholera, and smallpox frequently broke out. In some years, there were as many as 40,000 cases of smallpox, 9.5 million cases of measles, and a total of 12 million cases of schistosomiasis. Infectious diseases ranked first among all causes of death. In the early 1990s, cholera had serious outbreaks in some local areas. During the 1980s and early 1990s, epidemic hemorrhagic fever and rabies were rampant, and reported cases of hepatitis B continued to rise. From the end of 1988 to the beginning of 1989, a large-scale hepatitis A outbreak occurred in the Shanghai area, infecting over 300,000 people in just over a month (18). From 2018 to 2023, the Chinese government provided special subsidies for the prevention and control of major infectious diseases, incorporating this into the government’s annual budget expenditure projects. In 2018, the investment in infectious disease prevention and control reached 16,981.77 billion RMB, and by 2023, it had increased to 23,820.01 billion RMB, showing a trend of annual growth in the investment amount. Meanwhile, the government has strengthened the monitoring and early warning systems for public infectious diseases and implemented various measures such as vaccination, health education, public awareness campaigns, and medical interventions to control the spread of public infectious diseases. In recent years, the incidence of Class A, B, and C infectious diseases has been controlled at an average of 400–500 cases per 100,000 people, and the mortality rate has been greatly reduced.

China’s control of the incidence of public infectious diseases is similar to that of other developing countries. In some developing countries, the lack of medical resources and inadequate public health systems means that infectious disease outbreaks often lead to the overloading of healthcare systems, resulting in high mortality rates. For example, in Sub-Saharan African countries, the rise in the incidence of infectious diseases like AIDS and tuberculosis has led to a significant increase in mortality. Although China’s medical conditions have improved, rising incidence rates still have a major impact on mortality rates. In contrast, developed countries demonstrate stronger control over infectious diseases. Europe and North America, through comprehensive vaccination programs, disease monitoring, and early intervention mechanisms, are able to prevent increases in incidence from translating into high mortality rates. The proportion of funding investment has a significant negative impact on mortality, indicating that increased funding helps reduce mortality rates, a pattern observed globally. Developed countries have higher public health prevention expenditure ratios, such as the United States and Germany, where the proportion of public health prevention funding is relatively high, effectively controlling mortality from infectious diseases. The U.S. Centers for Disease Control and Prevention (CDC) invests substantial funds annually in the prevention and control of infectious diseases to ensure a rapid response to outbreaks. The U.S. per capita public health expenditure on preventive care varies from year to year, but estimates show that around $250 to $350 per person is spent annually on preventive healthcare services, including funding for vaccination, health checkups, and health education programs. This amount is significantly higher than in many developing countries (39).

China is a populous country, and population density is particularly high in the central and eastern regions, making the control of infectious disease outbreaks and transmission critically important. Although health and epidemic prevention departments at all levels in China have made significant efforts, controlling the incidence and mortality rates of public infectious diseases still faces many challenges. From 2018 to 2023, the mortality rates per 100,000 population for Class A, B, and C infectious diseases were 1.67, 1.81, 1.54, 1.57, 1.55, and 1.82, respectively, while the incidence rates per 100,000 population were 556.89, 731.72, 405.14, 440.2, 470.9, and 1359.86, respectively. Looking at the data, the mortality rate increased from 1.67 in 2018 to 1.81 in 2019, then decreased to around 1.5 from 2020 to 2023, before rising again to 1.82 in 2023. Similarly, the trend of incidence and mortality rates is similar, with the incidence rate increasing to 1359.86 in 2023. The increase in mortality and incidence rates in 2023 was primarily due to the widespread outbreak of influenza, mainly attributed to the mutation of the coronavirus and severe winter weather conditions (29). From this trend, it is evident that the control of infectious diseases is complex, with fluctuations occurring between different years. The fluctuation in infectious disease trends does not follow a single directional trend, indicating that the difficulty of infectious disease prevention and control is significant, and cannot be achieved by a single control measure alone.

This study selected several parameters, including the mortality rate, incidence rate, proportion of funding allocation, and proportion of professional technical personnel, for regression analysis. Although the incidence rate and mortality rate of infectious diseases are directly related, the mortality rate is also influenced by the medical institution’s treatment capacity. The regression coefficient of the incidence rate is 0.001, indicating a direct positive relationship with the mortality rate. However, the level of mortality rate is also affected by the capacity of medical institutions to provide treatment. The selection of the proportion of funding allocation and the proportion of professional technical personnel for analysis does not imply that only these two factors can control the fluctuation of infectious disease mortality and incidence. In reality, there are many factors involved in infectious disease control, including investment in prevention and control, increasing and involving professional technical personnel, establishing sound monitoring and warning systems, vaccination, personal protective measures, medical and health measures, health education and public awareness, international cooperation and information sharing, emergency plans, and crisis management measures, among others. Many of these measures cannot be quantified, and some lack available data indicators. Therefore, the study chose parameters such as the incidence rate, proportion of funding allocation, and proportion of professional technical personnel to participate in the analysis. The aim was to provide an analytical method and model for understanding the relationship between the development trend of public infectious diseases and control measures. It is hoped that such methods and models can be used to analyze the effectiveness of infectious disease control measures and further provide relevant measures to control the spread of infectious diseases.

## 6 Conclusion

Through the data analysis conducted above, it is evident that the incidence rate and mortality rate of public infectious diseases fluctuate between different years, indicating that the trend of public infectious diseases is not consistently decreasing or increasing in the same direction. This fluctuation is attributed to various factors causing the onset of public infectious diseases, including social and natural environments, pathogen infections, and individual poor hygiene habits and lifestyles, among others. Many of these disease-causing factors are not easily controllable. Although substantial preventive and control measures have been implemented by society and government agencies, the prevention and control of public infectious diseases constitute a long-term and continuous process. On the other hand, there are numerous prevention and control measures for public infectious diseases. However, factors such as the number of professional technical personnel may not significantly contribute to controlling the spread of infectious diseases. Therefore, controlling the transmission trend of public infectious diseases requires the comprehensive utilization of various prevention and control measures to achieve effective prevention and control.

## Data Availability

The original contributions presented in the study are included in the article/Supplementary material, further inquiries can be directed to the corresponding author.
