# Supplementary material for: Analysis report on trends in public infectious disease control in China
Source: Front Public Health. 2025 Jan 7;12:1423191. doi: 10.3389/fpubh.2024.1423191 (PMC11746904; doi:10.3389/fpubh.2024.1423191)
Supplement: Supplementary file 1 [file Table_1.docx]

**Appendix 1**

Table 1. Number of reported incidences and deaths of Category A and B infectious diseases in the country from 2018 to 2023. Unit: Individual.

| Disease name | Number of cases | | | | | | death toll | | | | | |
| --- | --- | --- | --- | --- | --- | --- | --- | --- | --- | --- | --- | --- |
|  | 2018 | 2019 | 2020 | 2021 | 2022 | 2023 | 2018 | 2019 | 2020 | 2021 | 2022 | 2023 |
| plague |  | 5 | 4 | 1 | 2 | 5 | 0 | 1 | 3 |  | 1 | 1 |
| cholera | 28 | 16 | 11 | 5 | 31 | 29 | 0 |  |  |  |  |  |
| Infectious SARS |  |  |  |  |  | 0 | 0 |  |  |  |  |  |
| AIDS | 64170 | 71204 | 62167 | 60154 | 52058 | 59533 | 18780 | 20999 | 18819 | 19623 | 18885 | 20289 |
| viral hepatitis | 1280015 | 1286691 | 1138781 | 1226165 | 1105865 | 1674252 | 531 | 575 | 588 | 520 | 543 | 2147 |
| polio |  |  |  |  |  |  |  |  |  |  |  |  |
| Avian Influenza |  |  |  |  | 1 | 1 |  |  |  |  |  |  |
| measles | 3940 | 2974 | 856 | 552 | 552 | 950 | 1 |  |  |  |  |  |
| epidemic hemorrhagic fever | 11966 | 9596 | 8121 | 9187 | 5218 | 5549 | 97 | 44 | 48 | 64 | 34 | 14 |
| Rabies | 422 | 290 | 202 | 157 | 133 | 131 | 410 | 276 | 188 | 150 | 118 | 127 |
| Japanese encephalitis | 1800 | 416 | 288 | 207 | 146 | 235 | 135 | 13 | 9 | 6 | 3 | 6 |
| dengue | 5136 | 22188 | 778 | 41 | 547 | 19627 | 1 | 3 |  |  |  | 1 |
| anthrax | 336 | 297 | 224 | 392 | 349 | 465 | 3 | 1 |  | 2 | 2 | 2 |
| Bacillary and amoebic dysentery | 91152 | 81075 | 57820 | 50403 | 35951 | 37425 | 1 | 1 | 2 | 3 | 1 | 2 |
| tuberculosis | 823342 | 775764 | 670538 | 639548 | 560847 | 773512 | 3149 | 2990 | 1919 | 1763 | 2205 | 2883 |
| Typhoid and paratyphoid | 10843 | 9274 | 7011 | 7244 | 5829 | 5749 | 2 |  | 5 |  | 2 | 1 |
| meningococcal meningitis | 104 | 111 | 50 | 63 | 59 | 99 | 10 | 6 | 3 | 5 | 5 | 1 |
| pertussis | 22057 | 30027 | 4475 | 9611 | 38295 | 38205 | 2 | 2 | 1 | 2 | 2 | 4 |
| diphtheria |  |  | 2 |  |  | 1 |  |  |  |  |  |  |
| neonatal tetanus | 83 | 65 | 34 | 23 | 19 | 22 | 4 | 5 | 1 | 1 | 1 |  |
| scarlet fever | 78864 | 81737 | 16564 | 29503 | 20794 | 25276 |  |  | 1 |  |  |  |
| Brucellosis | 37947 | 44036 | 47245 | 69767 | 66138 | 75858 |  | 1 |  | 3 |  | 3 |
| gonorrhea | 133156 | 117938 | 105160 | 127803 | 96313 | 106233 | 1 |  |  |  | 1 | 0 |
| syphilis | 494867 | 535819 | 464435 | 480020 | 441159 | 616933 | 39 | 42 | 54 | 30 | 23 | 33 |
| leptospirosis | 157 | 214 | 297 | 403 | 190 | 308 | 1 | 2 | 8 | 2 | 2 |  |
| Schistosomiasis | 144 | 113 | 43 | 13 | 13 | 38 |  |  |  |  |  |  |
| malaria | 2518 | 2487 | 1051 | 780 | 820 | 2406 | 6 | 19 | 6 | 3 | 6 | 11 |
| H7N9 | 2 | 1 |  |  |  | 0 | 1 | 1 |  |  |  |  |
| Monkeypox |  |  |  |  |  | 64963 |  |  |  |  |  |  |
| total | 3063049 | 3072338 | 3072338 | 2586157 | 2431329 | 3507805 | 23174 | 24981 | 24981 | 21655 | 22177 | 25525 |

Data source: Chinese Health Commission and Chinese National Administration of Disease Control and Prevention website [23,24,25,26,27,28].
